# Do Harbour Porpoise Mortality Records Reflect Living Population Structure? A Matrix Population Model Diagnostic

**DOI:** 10.1002/ece3.74607

**Published:** 2026-11-01

**Authors:** Michaela Kirstine S. M. H. Søgaard, Magnus Wahlberg, Owen R. Jones

**Affiliations:** ^1^ Department of Biology University of Southern Denmark Odense Denmark; ^2^ Marine Biological Research Centre University of Southern Denmark Kerteminde Denmark; ^3^ Fjord & Bælt Kerteminde Denmark

## Abstract

Effective conservation of marine mammals depends on reliable demographic information, yet acquiring such data for highly mobile cetaceans is challenging. Harbour porpoises ( *Phocoena phocoena* ) are widely used as sentinel species, but much of what is known about their demography comes from stranding and bycatch records. While invaluable, these data may be filtered by drift, detection, reporting effort and age‐specific vulnerability. We developed a matrix population model (MPM)‐based diagnostic framework to quantify how observed stage compositions in mortality datasets deviate from asymptotic demographic expectations. Using a stage‐structured MPM parameterised from published vital rates, we derived the stable stage distribution (SSD) and compared it with age‐class distributions from 10,863 harbour porpoise strandings (of 16,181 total records) in Danish and North Sea waters, bycatch and mixed‐mortality records, and hunting captures from Greenland and Denmark. Deviations from SSD, and from the composition expected among deaths, were assessed using goodness‐of‐fit tests, distance metrics, and tests of the juvenile proportion. Strandings showed strong and consistent departures from SSD expectations, with juveniles markedly over‐represented across spatial and temporal scales. Juveniles remained over‐represented relative to the composition expected among deaths under baseline rates, and the departure from the living population was robust to variation in vital rates. Divergence from the asymptotic stage structure was moderate to strong, varied across space, and increased significantly over time in 2 of 6 regions. Bycatch, mixed‐mortality, and hunting captures showed comparable SSD divergence, indicating that juvenile‐heavy mortality composition is not an artefact of stranding sampling alone. Our results show that harbour porpoise stranding, bycatch, and even hunting data should not be assumed to be demographically representative of living populations. Separating sampling bias from higher juvenile mortality needs independent estimates of juvenile survival.

## Graphical abstract

Using a matrix population model–based diagnostic, we show that age structures from harbour porpoise strandings, bycatch, and hunting records deviate from demographic expectations, with juveniles strongly over‐represented. These findings demonstrate that commonly used mortality datasets are not demographically representative of living populations and should be treated as potentially selectively biased in conservation and demographic analyses.

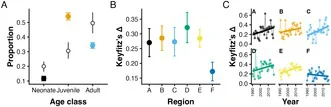

## 1 Introduction

The ability to monitor and manage wild populations hinges on reliable demographic information, including knowledge of survival, reproduction, and population structure across age or stage classes. For marine mammals, and cetaceans in particular, obtaining such data presents formidable challenges. These animals are highly mobile, spend most of their time beneath the surface and out of direct view, and are distributed across expansive and often inaccessible marine habitats. Systematic demographic studies typically require dedicated surveys at considerable logistical and financial cost, and even these efforts provide only snapshot views of population status at specific times and locations.

Against this backdrop, opportunistic data sources become critically important. Chief among these are stranding and bycatch records: observations of dead or dying animals that wash ashore or are caught in fishing nets, and are then reported, recovered, and examined. Strandings and bycatch records have long been recognised as valuable windows into cetacean biology, providing material for studies of distribution, diet, reproductive status, contaminant burdens, disease, and cause of death (e.g., Kesselring et al. 2017; Kinze et al. 2021; Peltier et al. 2013; Siebert et al. 2020). However, the extent to which stranding and bycatch data can reliably inform our understanding of demographic processes in living populations remains an open question. The fundamental concern is representativeness: do the animals that strand or are bycaught reflect the age structure, sex ratios, and vital rates of the populations from which they come, or are certain demographic groups systematically over‐ or under‐represented?

Multiple mechanisms could introduce demographic bias into stranding and bycatch datasets, if their effect differs among age classes, so that population parameters derived from them do not represent the wild population. Carcass drift, driven by ocean currents, wind, and the buoyancy of decomposing bodies, may transport animals far from their sites of death, creating a spatial mismatch between mortality and recovery (Peltier et al. 2013). Detection probability varies with coastline accessibility, human population density, and dedicated search effort, introducing sampling heterogeneity that is rarely quantified. Reporting effort fluctuates across regions and over time, influenced by public awareness campaigns, institutional capacity and changing social priorities. Perhaps most importantly, different age classes may exhibit differential vulnerability to mortality and recovery. Young animals, particularly neonates and juveniles, may be more susceptible to environmental stressors, predation, and starvation, and their smaller size and reduced blubber stores could affect buoyancy and drift behaviour. Young animals may also be naive when encountering fishing gear for the first time. Conversely, older adults might be under‐represented if they die in offshore waters, where carcasses sink before reaching shore, and prior exposure to fishing gear may reduce their susceptibility to entanglement.

The harbour porpoise ( *Phocoena phocoena* ) is ideal for investigating these questions. It is the most abundant cetacean in European shelf waters, with high densities in the North Sea and along Danish coasts (Hammond et al. 2013; Scheidat et al. 2008). Porpoises across the North Atlantic, including the North Sea, are largely one population with some isolation by distance and some sub‐structure in the southern North Sea, and West Greenland may hold a separate population (Olsen et al. 2022; Celemín et al. 2025; Autenrieth et al. 2024). The Belt Sea population is considered separate (Owen et al. 2024). The abundance of porpoises in the Baltic Sea proper is substantially lower and conservation concern correspondingly higher (Cervin et al. 2020). Harbour porpoises face a range of anthropogenic pressures, including habitat degradation, incidental capture in fisheries (bycatch), chemical and acoustic pollution, prey depletion and climate‐driven ecosystem changes. Given these multiple stressors, robust demographic monitoring is essential for assessing population trends and informing management decisions.

Harbour porpoise life history is typical of marine mammals: a relatively long lifespan, delayed maturity, low annual reproductive output, and limited capacity for rapid population recovery after decline. Females typically reach sexual maturity between 3 and 5 years of age and produce a single calf annually or biennially (Møhl‐Hansen 1954; Clausen and Andersen 1988; Lockyer 2003; Lockyer and Kinze 2003). First‐year mortality is elevated, as in many mammal species, and survival increases with age through the juvenile period before stabilising in adulthood.

Despite the ecological and conservation importance of this species, systematic demographic surveys remain spatially and temporally limited. Much of the available information on population structure, trends, and mortality patterns comes from stranding records. Notably, IJsseldijk et al. (2020) compiled and analysed more than 16,000 harbour porpoise stranding records from the North Sea region spanning nearly three decades (1990–2017). Their comprehensive study documented spatiotemporal patterns in stranding frequency, identified seasonal peaks in neonatal strandings, described variation in sex and age structure across regions, and highlighted biologically meaningful geographic heterogeneity in mortality signals. However, their analytical focus was on describing and modelling stranding patterns rather than on formally testing whether the demographic composition of strandings reflects that of living populations.

Our study addresses this complementary question. Rather than modelling stranding frequency or spatial distribution, we ask whether the observed age‐class composition across multiple harbour porpoise mortality datasets (strandings, bycatch records and hunting captures) is consistent with theoretical expectations derived from population ecology. To do so, we employ matrix population models (MPMs), a well‐established framework for describing and projecting the dynamics of stage‐ or age‐structured populations (Caswell 2001). MPMs organise demographic information—survival, growth and reproduction—into a projection matrix that governs how population size and structure evolve over discrete time intervals. A central property of any ergodic MPM is the stable stage distribution (SSD): the asymptotic proportional representation of each stage class under the assumed vital rates. We therefore use SSD as a mechanistic reference expectation for mortality samples. Under the assumed demography, age‐specific mortality is part of the biological process that gives rise to the expected stage structure; the critical sampling question is whether post‐mortem recovery processes such as drift, detection, and reporting are approximately non‐selective with respect to age. If recovery is non‐selective, the composition of recovered mortality samples would be expected to approach SSD. Departures from SSD therefore indicate selective demographic filtering and/or a mismatch with asymptotic assumptions. Because natural populations can show transient structure under environmental variability and episodic recruitment, SSD is treated here as a reference benchmark rather than as a claim about the realised population state.

We predict, based on documented early‐life vulnerability in marine mammals and terrestrial ungulates (Cook et al. 1971; Linnell et al. 1995), that juveniles will be systematically over‐represented in mortality records relative to SSD expectations, and that this pattern will be detectable across data types despite uncertainty in model parameterisation. A secondary prediction is that strandings will show greater demographic bias than bycatch, because the processes linking death and recovery are more indirect and spatially displaced in strandings than in direct bycatch observation. Whether bias differs further between bycatch and hunting captures remains an open question. The spatial and temporal structure of bias within the stranding record is less predictable: whether divergence is consistent across regions, or varies in ways that reflect localised differences in mortality pressure, environmental conditions or reporting effort. Quantifying this heterogeneity and assessing whether it has changed over the nearly three‐decade stranding record are additional empirical targets.

Our analytical approach comprises two complementary components. First, we conduct hypothesis tests and compute distributional distance metrics to assess divergence between observed stage compositions across strandings, bycatch, and hunting records and SSD expectations, with particular attention to juvenile over‐representation. Second, we implement sensitivity and robustness analyses to evaluate how strongly our conclusions depend on model assumptions, parameterisation choices and data completeness. We treat published vital rates as uncertain assumptions, not fixed values, and this includes varying them over wide ranges, propagating uncertainty in SSD estimates through simulation, testing the impact of missing age classes and low sample sizes, and employing alternative distance metrics to ensure inferential stability.

Throughout, ‘demographic bias’ means a departure of the observed stage composition from that of the living population; it does not by itself show whether the cause is sampling bias or simply higher mortality at some stages.

By explicitly quantifying demographic bias and rigorously propagating uncertainty, we aim to provide both a substantive answer for harbour porpoises and a reusable analytical template for systems where representativeness is uncertain. The framework requires only two inputs: stage‐composition data from mortality sources (e.g., strandings or bycatch) and demographically informed priors on vital rates. Both are increasingly available for species of conservation concern. The result is an explicit, quantitative diagnostic of departure from asymptotic demographic expectations that can inform how mortality records should be weighted when making inferences about living populations.

## 2 Methods

### 2.1 Study Area and Data Sources

Our study draws on harbour porpoise mortality data from across the North Atlantic and adjacent seas—including the North Sea, the Belt Sea, and the western North Atlantic off Greenland—with the primary analytical focus on the North Sea, where the stranding data come from; the Belt Sea region is represented by the Møhl‐Hansen hunting data and the bycatch data from the south‐western Baltic. This study area is a biogeographically complex mosaic of habitats, varying in depth, salinity, primary productivity, and intensity of anthropogenic use, spanning multiple management jurisdictions and hosting genetically differentiated harbour porpoise populations. We draw on three classes of mortality data: stranding records from coordinated North Sea monitoring networks, bycatch and mixed‐mortality records from studies covering Dutch, Irish, German, Danish, Norwegian, and UK waters, and historical hunting captures from two sources including Funen, Denmark and West Greenland. These sources differ in geographic scope, sampling period and collection method.

The primary stranding dataset is compiled and described by IJsseldijk et al. (2020), comprising nearly three decades of coordinated monitoring across multiple national stranding networks. The dataset includes records from Denmark, Germany, the Netherlands, Belgium, the United Kingdom, and other North Sea states. For each stranded individual, observers recorded morphometric measurements (total body length, girth, condition), sex, estimated decomposition state, and where possible, age. Age was determined from tooth growth layer counts or, where these were unavailable, assigned from body length using established life‐history criteria. We focus on individuals with available body length measurements, as length serves as a reliable and operational proxy for demographic stage in this species.

Bycatch records were drawn from four studies: Brennecke et al. (2021) from the south‐western Baltic, Frie and Lindstrøm (2024) from Norwegian waters, IJsseldijk et al. (2022) from the Netherlands, and Murphy et al. (2020) from the Celtic/Irish Sea and North Sea coast of the UK. These vary in sampling period, geographic coverage, and the form in which age or stage information is reported. Importantly, the Murphy North Sea management‐unit component partially overlaps with the IJsseldijk et al. (2020) stranding coverage (regions A‐C and part of D), so this source is treated as partially non‐independent in interpretation. Historical hunting captures come from two sources: Møhl‐Hansen (1954), based on drive hunts at Gamborg Fjord, Funen, Denmark between 1941 and 1944, and Lockyer et al. (2001), based on catches from West Greenland between 1988 and 1995. Full details of sample sizes, sampling units, and stage‐harmonisation procedures for all sources are given in Table 1.

**TABLE 1:** Mortality data sources used for stage‐structure comparison against SSD.

Data type	Source	Site/Stratum	Years	Unit type	*n* (used)	Stage mapping
Strandings	IJsseldijk et al. (2020)	North Sea regions A‐F	1990–2017	Individual‐level records	10,863	Source‐defined length‐based Neonate/Juvenile/Adult classes
Bycatch	Brennecke et al. (2021)	South‐western Baltic Sea	Oct 1987–Nov 2016 (all months represented)	Individual‐level records	136	Stage labels harmonised to Neonate/Juvenile/Adult
Bycatch	Frie and Lindstrøm (2024)	Norway	Autumn 2016; Spring 2017	Individual‐level records	134	Age‐to‐stage mapping (< 1, 1 to < 4, ≥ 4 years)
Bycatch	IJsseldijk et al. (2022)	Netherlands	2008–2019 (all months)	Aggregate percentages (bycatch‐only subset)	103	Reported Neonate/Juvenile/Adult proportions
Mixed (bycatch + strandings)	Murphy et al. (2020)	Celtic/Irish Sea (1990–1999)	1990–1999	Counts read from published Figure 2 (mixed bycatch + strandings)	217	Counts read from published Figure 2 (*n* = 645), age‐bin lower bounds mapped to stages
Mixed (bycatch + strandings)	Murphy et al. (2020)	Celtic/Irish Sea (2000–2012)	2000–2012	Counts read from published Figure 2 (mixed bycatch + strandings)	172	Counts read from published Figure 2 (*n* = 645), age‐bin lower bounds mapped to stages
Mixed (bycatch + strandings)	Murphy et al. (2020)	North Sea (1990–1999)	1990–1999	Counts read from published Figure 2 (mixed bycatch + strandings)	155	Counts read from published Figure 2 (*n* = 645), age‐bin lower bounds mapped to stages
Mixed (bycatch + strandings)	Murphy et al. (2020)	North Sea (2000–2012)	2000–2012	Counts read from published Figure 2 (mixed bycatch + strandings)	101	Counts read from published Figure 2 (*n* = 645), age‐bin lower bounds mapped to stages
Hunting	Lockyer et al. (2001)	Maniitsoq and northerly sites	Jul–Sep 1988–1989; 1995	Aggregate counts	126	Age‐year counts mapped to stages
Hunting	Lockyer et al. (2001)	Nuuk and Paamiut	Jul–Sep 1988–1989; 1995	Aggregate counts	52	Age‐year counts mapped to stages
Hunting	Møhl‐Hansen (1954)	Denmark (hunting captures)	Nov–Feb 1941–1944	Aggregate counts	389	Length thresholds (< 91, 91–130, > 130 cm)

*Note:* This shows the provenance, sampling unit, sample size, and stage‐harmonisation approach for each source included in the comparative analyses.

### 2.2 Stage‐Structured Matrix Population Model

We developed a female‐only, stage‐structured matrix population model for harbour porpoise based on a post‐breeding annual census. Because the observational data include both sexes, this introduces a minor mismatch; however, if sex ratios in mortality datasets are approximately 1:1, the female‐only SSD provides a close approximation to the all‐sex stage structure, and departures from equal sex ratios would not alter the directional conclusion of juvenile over‐representation. The population was divided into three life‐history stages: neonates (age < 1 year), juveniles (age ≥ 1 and < 4 years) and adults (age ≥ 4 years). These boundaries align with published growth and maturity schedules—derived from biological sampling including bycatch, directed catches and strandings—for North Sea porpoises (Lockyer 2003; Lockyer and Kinze 2003), and are consistent with stage groupings commonly used in stranding‐based demographic analyses (IJsseldijk et al. 2020).

Stage dynamics follow $n_{t+1}=An_{t}$, with Lefkovitch projection matrix:

$$A=\left[\begin{array}{ccc} 0 & 0 & F_{A}\\ S_{0} & P_{J} & 0\\ 0 & G_{J} & P_{A} \end{array}\right]$$

where $F_{A}$ is female neonate production per adult female per year, $S_{0}$ is survival from birth to age 1, P denotes stage stasis (remaining in a stage), and G denotes forward transition to the next stage. Adult natural survival was set to $S_{A}=0.867$, the annual survival rate from the Danish life table of Kinze (1990) as reported by Lockyer (2003), and adult stasis was therefore set to $P_{A}=S_{A}$.

Neonate survival (birth to age 1) was parameterised using the square rule of Woodley and Read (1991), $S_{0}=S_{A}^{2}=0.752$, to reflect elevated calf vulnerability and potential mortality associated with maternal loss. Woodley and Read (1991), citing Reilly and Barlow (1986), present this formulation as an upper‐bound heuristic for first‐year survival and note a lower bound near 0.5 based on values typical of pinnipeds and long‐lived terrestrial mammals. We adopt this upper‐bound value as a conservative baseline—one that, if anything, understates early‐life mortality and therefore makes detected juvenile over‐representation harder to explain by model parameterisation alone.

Juvenile progression was modelled with a constant maturation probability (g) conditional on survival, such that $G_{J}=S_{J}g$ and $P_{J}=S_{J}\left(1-g\right)$. Setting g=1/3 yields an expected juvenile‐stage duration of 3 years (ages 1–3), consistent with maturation around age 4 under a Lefkovitch stage‐structured formulation (Caswell 2001). With baseline juvenile survival $S_{J}=0.81$ (the midpoint of $S_{0}$ and $S_{A}$, because juvenile survival is expected to lie between first‐year and adult survival), this gives $G_{J}=0.27$ and $P_{J}=0.54$. This implies a geometric distribution of juvenile duration (mean three years); we test a fixed‐duration alternative in the Supplement (see Section 2.7).

Fertility was parameterised as $F_{A}=b\;p_{f}$, where b is calves per adult female per year and $p_{f}=\frac{1}{1+r}$ is the female birth fraction derived from the male: female sex ratio r. Assuming single‐calf reproduction, we interpret pregnancy rate as expected calves per adult female per year. Using the foetal sex ratio reported by Lockyer (2003) (r=1.1–1.2), which we treat as a proxy for sex ratio at birth, we used the midpoint approximation $p_{f}=0.466$, giving baseline fecundity $F_{A}=0.90\times 0.466=0.419$.

Under this annual post‐breeding formulation the neonate stage lasts exactly one projection interval, so neonate stasis is zero (the top‐left element of the matrix) and neonate survival ($S_{0}$) is the forward transition, so that all surviving neonates advance to the juvenile stage by the next census.

With these baseline values the model has a population growth rate of λ=1.03, that is, a population growing by about 3% a year, which is within the 5%–10% maximum reported for porpoises (Lockyer 2003). Because the true growth rate of the populations sampled is not known, we also report results with the model adjusted to λ=1 (see Section 2.7).

Stage‐specific natural survival beyond the first year is uncertain and varies among regions and sampling sources; we therefore treat juvenile survival ($S_{J}$) as an explicit model assumption, constrained between neonate and adult survival, and evaluated through sensitivity analyses. More generally, we treat published vital rates as uncertain assumptions, not fixed values: they differ among populations and may themselves be biased if they come from opportunistic samples, and a central aim of this study is to show how much our conclusions depend on them. We parameterise a baseline demographic model intended to approximate natural demographic processes, recognising that published estimates may still reflect some anthropogenic impacts (Table 2).

**TABLE 2:** Baseline matrix population model parameter values, descriptions, and sources used for SSD estimation.

Parameter	Value	Description	Source/Justification
*F*_A_	0.419	Female neonates per adult female per year (matrix element)	Derived as *F*_A_ = *b* × *p*_*f*_ = 0.90 × 0.466
*P*_J_	0.540	Juvenile stasis probability (matrix element)	Derived as *P*_J_ = *S*_J_ (1 − *g*) with *S*_J_ = 0.81 and *g* = 1/3
*G*_J_	0.270	Juvenile‐to‐adult transition probability (matrix element)	Derived as *G*_J_ = *S*_J_ × *g* with *S*_J_ = 0.81 and *g* = 1/3
*P*_A_	0.867	Adult stasis probability (matrix element)	Set equal to adult survival (*P*_A_ = *S*_A_)
*S*_A_	0.867	Adult annual natural survival	Annual survival from the Danish life table of Kinze (1990), as reported by Lockyer (2003)
*S*_0_	0.752	Neonate (0–1 year) survival; also the neonate‐to‐juvenile transition in the matrix (neonate stasis is zero because the neonate stage lasts one year)	Square rule (*S*_0_ = *S*_A_ × *S*_A_); Woodley and Read (1991), citing Reilly and Barlow (1986); qualitative support in Lockyer (2003)
*S*_J_	0.81	Juvenile annual survival	Assumed (midpoint of *S*_0_ and *S*_A_); constrained by *S*_0_ < *S*_J_ ≤ *S*_A_; tested via sensitivity analyses
*g*	1/3	Annual maturation probability	Derived from juvenile duration (ages 1–3); Lockyer (2003); Lockyer and Kinze (2003)
*b*	0.90	Calves per adult female per year	Baseline value within reported pregnancy‐rate range (0.74–0.986); Lockyer (2003)
*p*_*f*_	0.466	Female birth fraction	*p*_*f*_ = 1/(1 + *r*), with Lockyer (2003) foetal sex ratio *r* = 1.1–1.2 (male: female), midpoint approximation

### 2.3 Statistical Comparison of Observed and Expected Stage Distributions

Unless stated otherwise, ‘expected’ refers to the stable stage distribution (SSD), which describes the living population; the expected composition of deaths is introduced in a later subsection.

To assess whether observed stage distributions in strandings differ from SSD expectations, we employed a multi‐faceted statistical approach that combined formal hypothesis tests, distributional distance metrics and targeted directional tests. Observed stage distributions were computed at multiple aggregation levels: overall (pooling all strandings across space and time), by region, by year, and by region‐year combinations. For each grouping, we calculated the proportions of neonates, juveniles and adults.

Our primary statistical tests and metrics were a two‐sided proportion test for the juvenile proportion and Keyfitz's Δ.

#### 2.3.1 Proportion Test for Juveniles

Motivated by a priori expectations of elevated early‐life mortality, we tested whether the observed proportion of juveniles differs from the expected proportion. This was implemented as a one‐sample, two‐sided proportion test of the null hypothesis that $p_{\text{juvenile}}=p_{\text{expected},\text{juvenile}}$. We use a two‐sided test for three reasons: the aim is to detect bias in either direction, and a juvenile deficit would be as informative as an excess; the direction relative to the SSD is not fixed in advance, because the model already includes higher early‐life mortality; and the test is applied to many sources, for which a pre‐specified direction is hard to justify.

#### 2.3.2 Keyfitz's Δ

This metric quantifies the overall magnitude of divergence between observed and expected distributions. Values range from 0 (perfect match) to 1 (complete mismatch). Keyfitz's Δ has an intuitive interpretation: it represents the minimum proportion of individuals that would need to be reassigned to different stages to make the observed distribution match the expected distribution (Keyfitz 1968; Jones 2021). Values above 0.10–0.15 indicate departures of practical significance.

$$\Delta =\frac{1}{2}\;\sum_{i}\left|p_{i,\mathrm{obs}}-p_{i,\mathrm{SSD}}\right|$$

where $p_{i,\mathrm{obs}}$ and $p_{i,\mathrm{SSD}}$ are observed and expected stage proportions.

As concordance checks, we also computed chi‐square goodness‐of‐fit statistics and Hellinger distance. To keep the main text focused on Keyfitz's Δ, full definitions, equations and implementation details for these secondary diagnostics are provided in the Supporting Information (Supplementary Methods; Table S9).

To quantify and display uncertainty we used two distinct error‐bar definitions, depending on the plotted quantity. For stage‐proportion plots (e.g., observed Neonate/Juvenile/Adult proportions), we used normal‐approximation 95% confidence intervals for a binomial proportion:

$$\hat{p}\pm 1.96\sqrt{\frac{\hat{p}\left(1-\hat{p}\right)}{n}},$$

with bounds truncated to the feasible range [0, 1]. These intervals quantify sampling uncertainty in the estimated stage proportions for each source/group.

For Keyfitz's Δ summaries, we used non‐parametric bootstrap 95% intervals. Within each source/group, we resampled stage counts using multinomial draws of size n with probabilities equal to the observed stage proportions, recalculated Keyfitz's Δ for each of 2000 bootstrap replicates, and reported the 2.5th and 97.5th percentiles. These intervals therefore represent uncertainty in divergence from SSD, not uncertainty in SSD itself.

### 2.4 Comparison With the Expected Composition of Deaths

The SSD describes the living population, whereas strandings, bycatch and hunting records are animals that have died. If mortality differs among stages, stages will not be equally represented among the dead and the living. We therefore also calculated the stage composition expected among deaths in a population with a stable structure. For each stage we multiplied the proportion of the population in that stage (the SSD) by the probability that an animal in that stage dies during a projection interval (one minus the stage's survival, i.e., one minus the column sum of the survival and transition part of the matrix), and rescaled the three values to sum to one. In the baseline model, annual mortality is 0.248 for neonates, 0.190 for juveniles and 0.133 for adults. All comparisons (juvenile proportion test, Keyfitz's Δ and chi‐square tests) were made against both the SSD and this death‐based expectation.

### 2.5 Temporal and Spatial Analyses

The IJsseldijk et al. (2020) data permit temporal and spatial analysis. We conduct these analyses at the regional scale, using the same region coding (A–F) defined in that paper. In brief: Region A covers northeastern Scotland (Thurso to St Fergus), including Orkney; Region B spans from St Fergus to Newcastle; Region C spans from Newcastle to Great Yarmouth; Region D includes the remaining English coast, plus Belgium and the Dutch Delta area; Region E includes the mainland Netherlands and the Dutch Wadden area; and Region F includes the North Sea coasts of Schleswig‐Holstein (Germany) and Denmark.

Specifically, to assess whether demographic bias varies across space and time, we computed region‐year‐specific Keyfitz's Δ values and examined their temporal trends. For each region with sufficient multi‐year data, we fitted simple linear regressions of Keyfitz's Δ on year, testing for significant positive or negative trends in divergence over the study period. We also assessed regional synchrony by calculating pairwise Pearson correlation coefficients among region‐specific Keyfitz's Δ time series to determine whether regions show coordinated temporal patterns in demographic bias.

### 2.6 Bycatch and Hunting Data

To directly compare mortality datasets within a common framework, we repeated the SSD‐divergence workflow for each additional mortality source, standardising all records into the same three stage classes (Neonate/Juvenile/Adult). Where sources reported stage labels directly, we harmonised terminology (Calf→Neonate, Immature→Juvenile, Mature→Adult). Where sources reported individual ages or age bins, we mapped to stages using explicit thresholds: age < 1 year → Neonate; age 1 to < 4 years → Juvenile; age ≥ 4 years → Adult. We also harmonised sampling strata where needed, retaining source‐specific area/period groupings such as the Murphy et al. (2020) area‐by‐period strata. For IJsseldijk et al. (2022), stage composition was already provided as neonate/juvenile/adult percentages and was used directly for the bycatch‐only subset (*n* = 103). For Murphy et al. (2020), individual‐level data were not available, so we used age‐frequency counts (ages 0–23 years) that we read from the bars of their Figure 2 for each management unit and period (*n* = 645 aged animals, sexes combined). The total and the number of animals aged under 1 year matched those given in the paper, so reading error is likely to be very small. We could not separate stranded from bycaught animals. For Lockyer et al. (2001), we used the age‐structured counts reported in table 1 of that paper. For Møhl‐Hansen (1954), 389 measured animals were available for stage‐structure analysis.

We then repeated the main analyses against the same SSD reference used for strandings: juvenile proportion tests and Keyfitz's Δ, with secondary chi‐square checks reported in the Supporting Information. For source‐year units with low expected counts, Monte Carlo chi‐square *p*‐values were used in those supplementary diagnostics. To assess comparative divergence, we compared year‐level Keyfitz's Δ values between bycatch and strandings using a paired one‐sided Wilcoxon test (alternative: bycatch < strandings), restricted to years with minimum sample sizes in both datasets.

### 2.7 Sensitivity and Robustness Analyses

To evaluate the robustness of our conclusions, we conducted the following sensitivity analyses.

#### 2.7.1 Exclusion of Incomplete Age‐Class Records

We repeated all analyses after excluding region‐years in which one or more stage classes were entirely absent. This tests whether conclusions are sensitive to the way missing data are handled.

#### 2.7.2 Minimum Count Thresholds

We imposed minimum sample size requirements (e.g., ≥ 10 or ≥ 20 total strandings per group) and re‐evaluated key findings to ensure they are not artefacts of small‐sample instability.

#### 2.7.3 SSD Uncertainty Propagation

Because our baseline SSD is derived from a single MPM parameterisation, we propagated uncertainty in matrix elements via simulation. Using compute_ci function from the mpmsim R package (Jones 2025), we generated perturbed U and F matrices around the baseline model, recalculated SSD for each simulated matrix, and derived confidence bounds for SSD proportions. We then re‐evaluated juvenile over‐representation and Keyfitz's Δ summaries against these bounds. Figure S1 and Table S1 show the full uncertainty spread around SSD expectations, clarifying why the main conclusions remain robust to plausible vital‐rate variation.

#### 2.7.4 Sensitivity to Vital Rates

Published vital rates are uncertain, so we tested how much the expected composition depends on them. We drew 20,000 random sets of vital rates (Latin hypercube sampling), each value independently from a plausible range: adult survival 0.80–0.99, juvenile survival 0.60–0.99, neonate survival 0.50–0.99, pregnancy rate 0.60–1.00, and the annual probability that a juvenile matures 0.25–0.50. For each set we calculated the SSD and the death‐based expectation and compared them with the observed stranding composition, for all sets and for sets with λ between 0.95 and 1.05 or between 0.98 and 1.02. We also changed each rate on its own with the others at baseline (Tables S12 and S13).

#### 2.7.5 Population Growth Rate

We repeated the comparisons with the model adjusted to λ=1, by lowering either fertility (to 0.65 calves per adult female per year) or adult survival (to 0.83, with neonate survival recalculated from it using the square rule), with other rates at baseline (Table S11).

#### 2.7.6 Working Backwards From the Data

To see what the data would imply if they were an unbiased sample of deaths, we fitted adult, neonate and juvenile survival to each observed composition (maximum likelihood, with λ between 0.97 and 1.03). Three rates are fitted to two independent proportions, so we use this only to show how far survival would have to differ from published values, not as an estimate of mortality (Table S14).

#### 2.7.7 Fixed Juvenile Duration

A constant yearly maturation probability implies a wide spread of juvenile durations. To check whether this matters, we built an age‐by‐age model in which juveniles (ages 1–3) move up exactly one age per year and adults start at age 4, with survival and fertility as in the baseline model, and repeated it with adulthood starting at age 3 or 5 (Tables S15–S17).

### 2.8 Simulation‐Based Plausibility Checks

To address uncertainty in the baseline parameterisation without assuming a single fixed set of vital rates, we asked whether any biologically plausible MPM, regardless of specific parameter values, produces an SSD resembling the observed stranding composition. To address this, we used the mpmsim R package (Jones 2025) to generate 500 random 3‐stage Lefkovitch matrices (archetype 4: monotonic forward stage progression without retrogression), constrained only to plausible population growth rates (λ between 0.7 and 1.3). For each simulated matrix, we computed the SSD and Keyfitz's Δ relative to the observed overall stranding composition, then summarised the proportion of matrices meeting resemblance thresholds (Keyfitz's Δ ≤ 0.15, and more conservatively Keyfitz's Δ ≤ 0.10). In these matrices all survival, transition and fecundity values varied freely; the only constraint was on λ. Full simulation settings are provided in the Supplementary Methods; Figure S3 (Data S1) provides an additional PCA projection of this simulation space.

All analyses were performed in R version 4.3.0, using the popbio (Stubben and Milligan 2007), mpmsim (Jones 2025), tidyverse (Wickham et al. 2019) and ggplot2 (Wickham 2016) packages. Code and derived data are available in the project repository (see Data Availability Statement).

## 3 Results

We present results in two stages. The stranding dataset, being the largest and most spatially and temporally resolved, forms the primary focus; bycatch and hunting records are then analysed within the same framework to assess whether patterns of demographic bias are specific to strandings or general across mortality data types.

### 3.1 Overall Stage Distribution and Divergence From SSD

The SSD describes the living population, so this first comparison asks how the animals that died differ from the living population; the comparison with the expected composition of deaths follows below.

Among strandings with assigned age class (*n* = 10,863 of 16,181 total records), the observed stage distribution differed markedly from the SSD predicted by our baseline MPM. The overall observed composition was 13.2% neonates, 58.1% juveniles and 28.7% adults, compared with SSD expectations of 20.1%, 30.5% and 49.4%—a juvenile elevation of 27.6 percentage points and an adult deficit of 20.7 percentage points (Figure 1A). The two‐sided juvenile proportion test was highly significant (*p* < 0.001), and overall divergence was substantial (Keyfitz's Δ, the share of animals that would have to change stage for the observed and expected compositions to match = 0.276, bootstrap 95% CI 0.266–0.284), consistent with our prediction of systematic juvenile over‐representation.

**FIGURE 1:**
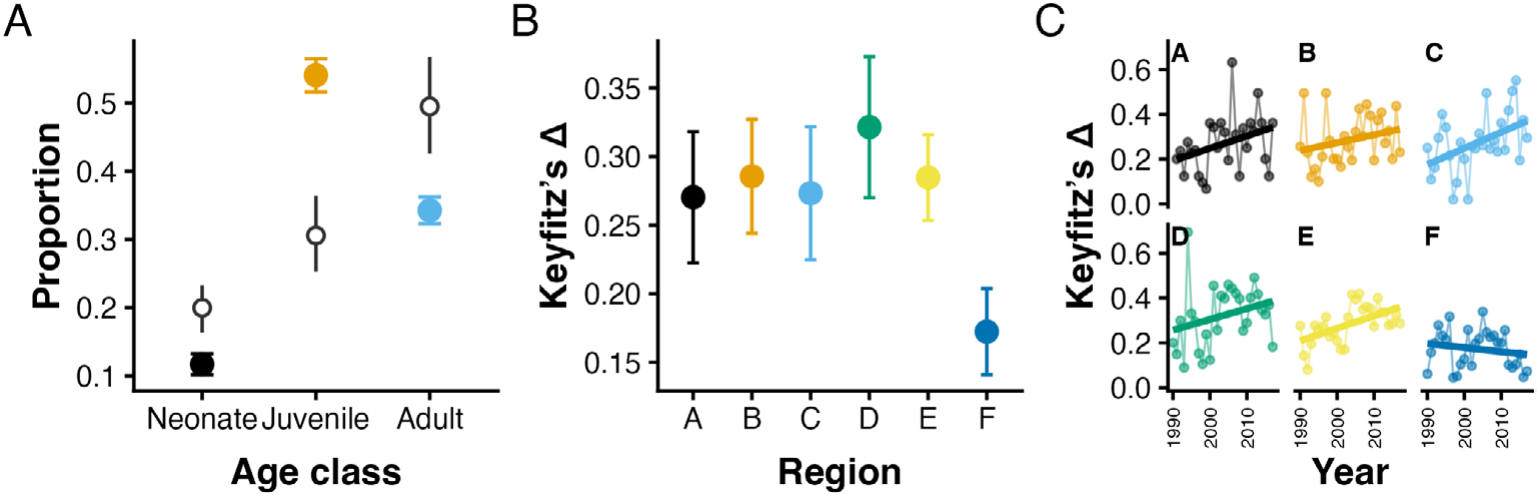
Stage composition in strandings differs systematically from SSD expectations, with divergence varying across regions and increasing over time in some areas. (A) Mean observed stage proportions across region‐years (Neonate, Juvenile, Adult) are shown as coloured points with 95% confidence intervals; SSD expected proportions and uncertainty intervals are overlaid for direct comparison. (B) Mean Keyfitz's Δ by strandings region (A–F), with 95% confidence intervals, summarises persistent regional differences in divergence from SSD. (C) Annual Keyfitz's Δ trajectories by region (facets A–F) with fitted linear trends show how SSD divergence changes through time within each region. References to sources of data are found in the text.

### 3.2 Simulation‐Based Plausibility of Alternative MPMs

To assess whether the observed mortality composition could plausibly arise under any reasonable demographic parameterisation, we generated 500 random biologically plausible 3‐stage Lefkovitch matrices. Only 5 (1.0%) produced SSDs within Keyfitz's Δ ≤ 0.15 of the observed stranding composition, and only 2 (0.4%) met the stricter Keyfitz's Δ ≤ 0.10 threshold. Most plausible matrices were therefore substantially farther from the observed composition (median Keyfitz's Δ = 0.557, range 0.075–0.832), indicating that the observed juvenile‐heavy mortality composition is inconsistent with demographic equilibrium across the broader space of plausible vital‐rate combinations, not merely under our specific baseline parameterisation (Figure S3).

Alternative metrics and tests (Hellinger distance and chi‐square diagnostics) were in broad agreement with Keyfitz's Δ and are reported in Tables S2, S3 and S9. Descriptive context for region‐year sampling structure and stage‐composition trajectories through time is provided in Table S4 and Figure S5. The full region‐year distribution of Keyfitz's Δ is shown in Figure S2 and summarised in Table S2.

**FIGURE 2:**
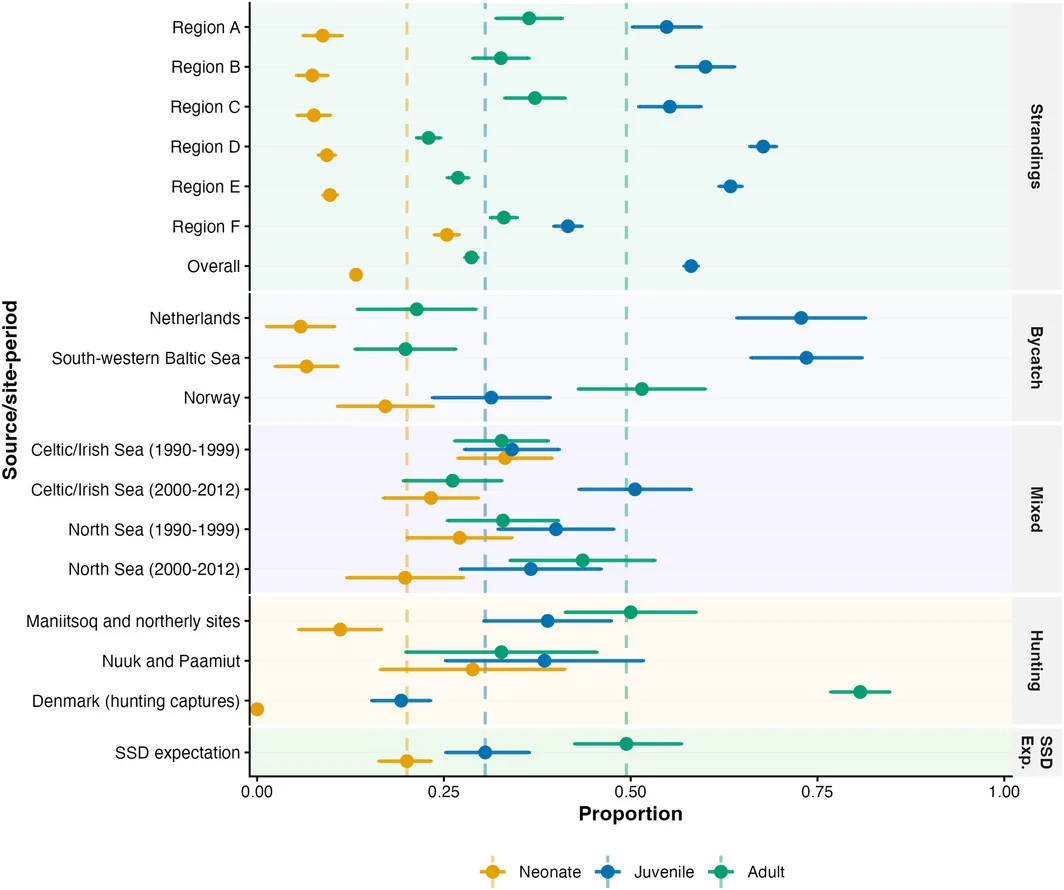
Strandings, bycatch, mixed‐mortality, and hunting age‐class proportions across sources, with SSD reference panel. Points are observed proportions by age class (Neonate/Juvenile/Adult) and horizontal error bars are 95% intervals for each source/site‐period. Rows are grouped into strandings, bycatch, mixed (bycatch + strandings), hunting, and SSD panels with light background shading to separate data types; the SSD panel shows expected proportions with uncertainty intervals from the SSD simulation. References for sources of data are found in the text.

**FIGURE 3:**
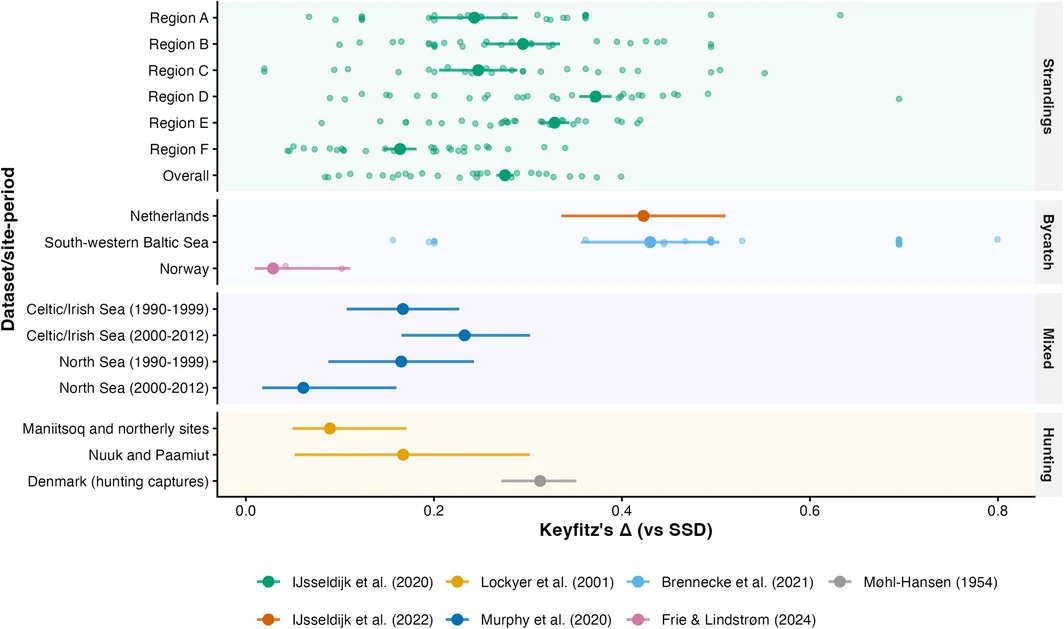
Combined SSD‐divergence comparison across strandings, bycatch and hunting sources. Points show Keyfitz's Δ estimates, and horizontal error bars show bootstrap 95% intervals. Faint jittered points indicate repeated year‐level values where available. Rows are grouped by data type (strandings, bycatch, mixed bycatch + strandings, hunting) with light background shading, and points are colour‐coded by source article (IJsseldijk et al. (2020, 2022), Lockyer et al. (2001), Murphy et al. (2020), Brennecke et al. (2021), Frie and Lindstrøm (2024), Møhl‐Hansen (1954)). Site and period labels are shown where relevant.

### 3.3 Comparison With the Expected Composition of Deaths

Among living animals, the baseline model expects 20.1% neonates, 30.5% juveniles and 49.4% adults; among deaths it expects 28.7%, 33.4% and 37.9%, because neonates and juveniles die at higher rates than adults. Strandings still contained far more juveniles than either expectation (Table S10): 58.1%, against 30.5% for the living population and 33.4% for deaths. Keyfitz's Δ fell from 0.28 (SSD) to 0.25 (deaths), and the juvenile proportion differed significantly from both (two‐sided *p* < 0.001), as it did in every region.

The two large bycatch samples (Brennecke et al. 2021; IJsseldijk et al. 2022) were 73%–74% juveniles and also differed from both references, and the Danish hunting sample (Møhl‐Hansen 1954) had a significant juvenile deficit. Most of the remaining bycatch, hunting and mixed‐mortality samples were consistent with the death expectation; the exception was the Murphy et al. (2020) Celtic and Irish Seas 2000–2012 sample, which still had more juveniles than expected (Table S10). Two smaller samples were close to significance but did not differ significantly under two‐sided tests (Greenland, Maniitsoq, against the SSD; Murphy et al. North Sea 1990–1999 against the death expectation).

### 3.4 A Stable Population Does Not Remove the Pattern

In the baseline model, the population grows at about 3% a year. Lowering fertility or adult survival so that the population is stable (λ=1) did not reduce the mismatch with strandings: Keyfitz's Δ was 0.31 and 0.27 against the SSD and 0.27 and 0.30 against the death expectation, respectively (baseline: 0.28 and 0.25; Table S11). A stable population needs fertility below the published pregnancy‐rate range (0.65, against 0.74–0.99) or adult survival of 0.83, within the range of published estimates (0.80–0.87), so our baseline probably overstates growth slightly.

### 3.5 Sensitivity to Vital Rates

Across the 20,000 random sets of vital rates, the expected juvenile share of the living population ranged from 14% to 44% and never reached the 58.1% seen in strandings (Table S12), including in sets with λ between 0.95 and 1.05 or between 0.98 and 1.02. The death‐based expectation was more variable (1%–92% juveniles), and 3%–8% of sets reached the observed juvenile share, generally those with low juvenile survival (median about 0.68). Changing one rate at a time (Table S13), the juvenile share of the living population stayed within 25%–35%, whereas that of deaths ranged from 6% to 76%.

If strandings were an unbiased sample of deaths, the best‐fitting model would have juvenile survival of 0.62 and adult survival of 0.89 (Table S14), the juvenile value well below the one we assumed (0.81). We read this as a sign that strandings are not an unbiased sample of deaths, not as an estimate of true survival, because the fit is not unique.

### 3.6 Fixing the Length of the Juvenile Period

In the age‐by‐age model with juveniles aged 1–3 years, the expected juvenile share was 34.2% for the living population and 37.2% for deaths (three‐stage model: 30.5% and 33.4%; Tables S15 and S16), and strandings still differed strongly from both (Keyfitz's Δ = 0.24 and 0.21; Table S17). The expected juvenile share depended more on the length of the juvenile period than on whether it was fixed: it was 27% (living) and 29% (deaths) if adults start at age 3, and 40% and 43% if they start at age 5. Strandings differed significantly from expectations in all cases.

### 3.7 Geographic Variation in Demographic Bias

Regional analyses revealed pronounced heterogeneity in divergence from SSD, with stable between‐region differences persisting over time (Figure 1B,C). Using the region coding of IJsseldijk et al. (2020), mean region‐year divergence was highest in Region D (0.321) and lowest in Region F (0.172), with the remaining regions falling within an intermediate range. Although between‐region differences are statistically clear, the absolute spread in regional mean Keyfitz's Δ is moderate (0.149 from lowest to highest mean) and should be interpreted alongside substantial within‐region year‐to‐year variation (median region‐specific SD = 0.119).

### 3.8 Sensitivity to Missing Age Classes and Low Counts

Applying stricter data filters did not materially alter the juvenile‐over‐representation result. Across scenarios (all data, complete age classes only, minimum *n* = 10, and both filters), the observed juvenile proportion remained at 0.581 (Table S5), while the expected SSD juvenile proportion remained 0.305 and two‐sided tests remained highly significant (all *p* < 0.001). Likewise, the magnitude of divergence was stable under filtering. Mean region‐year Keyfitz's Δ varied only from 0.256 to 0.268, and median Keyfitz's Δ from 0.256 to 0.258 across scenarios (Table S6), indicating that the central inference is not driven by missing classes or small‐*n* groups.

### 3.9 Sensitivity to SSD Uncertainty

When SSD uncertainty bounds were propagated into hypothesis testing, the expected juvenile proportion ranged from 0.300 to 0.313 across SSD scenarios (Table S7). The observed juvenile proportion remained at 0.581 and continued to exceed expectation under all bounds (all *p* < 0.001). Distance‐based results were similarly robust. Mean Keyfitz's Δ across region‐years remained high, ranging from 0.262 to 0.272, with medians from 0.252 to 0.262 (Table S8), confirming substantial mismatch even under conservative SSD assumptions.

### 3.10 Temporal Trends in Demographic Bias

Keyfitz's Δ varied over time (Table 3); only Regions C and E showed significant positive slopes in divergence from the SSD (C: 0.007; E: 0.006 Keyfitz's Δ units per year; both *p* < 0.05). Region B was positive but non‐significant (0.004, *p* = 0.179), and Region F was the exception with a near‐zero negative, non‐significant slope (−0.002, *p* = 0.370; Figure 1C). In practical terms, an increase of ~0.007 Keyfitz's Δ units per year implies an increase of about 0.189 over the 27‐year study period, relative to the baseline mean region‐year Keyfitz's Δ of 0.268.

**TABLE 3:** Per‐region temporal trends in SSD divergence (Keyfitz's Δ~Year). This shows which regions have increasing, decreasing or stable divergence over time.

Region	Slope	SE (slope)	*p*	*R* ^2^	*n*
A	0.006	0.003	0.078	0.119	27
B	0.004	0.003	0.179	0.068	28
C	0.007	0.003	0.015	0.207	28
D	0.005	0.003	0.141	0.081	28
E	0.006	0.002	0.003	0.299	28
F	−0.002	0.002	0.370	0.031	28

### 3.11 Regional Synchrony in Demographic Bias

Pairwise correlations among region‐specific Keyfitz's Δ time series were generally modest. The strongest positive correlations were A‐C (*r* = 0.464) and D‐E (*r* = 0.452). The full regional correlation matrix is provided in Figure S4.

### 3.12 Comparative Strandings, Bycatch and Hunting Results

Using the standardised bycatch dataset (four harmonised sources mapped to the shared Neonate/Juvenile/Adult framework), juvenile over‐representation remained pronounced (observed juvenile proportion = 0.526, SSD expectation = 0.305, two‐sided *p* < 0.001), and overall Keyfitz's Δ was 0.221. In paired year‐level comparisons, mean bycatch Keyfitz's Δ (0.331) exceeded mean strandings Keyfitz's Δ (0.199), and the one‐sided test for lower bycatch divergence was not supported (*p* = 0.979; Figures 2 and 3). Hunting captures from Møhl‐Hansen (1954) and Lockyer et al. (2001), converted to the same stage structure, occupied the same divergence range as the other mortality sources. Across all chi‐square goodness‐of‐fit tests, expected counts were high (minimum expected = 91.4), with no cells below 5, so sparse‐cell corrections were not required; full chi‐square results are provided in Table S9.

In summary, strandings and bycatch both differed significantly from SSD expectations, with juveniles over‐represented in both datasets. Bycatch showed divergence at least as great as strandings. The magnitude of bias varied considerably across stranding regions and over time.

## 4 Discussion

Our results provide clear and robust evidence that harbour porpoise mortality datasets show a systematic departure from asymptotic demographic expectations, as reflected by the SSD predicted by a stage‐structured matrix population model. Strandings showed strong SSD divergence, and bycatch data were not less biased away from SSD: in paired years, mean bycatch Keyfitz's Δ (0.331) exceeded mean strandings Keyfitz's Δ (0.199), with no support for lower bycatch divergence (one‐sided *p* = 0.979). Both data types should therefore not be treated as demographic cross‐sections of living populations.

The most striking and consistent signal is pronounced juvenile over‐representation relative to SSD expectations. In the baseline comparison, the observed juvenile proportion exceeded the SSD expectation by 27.6 percentage points, while the adult proportion was 20.7 percentage points below the SSD expectation. This pattern is evident in strandings across all spatial scales and in sensitivity analyses, and it is also evident in bycatch (0.526 observed vs. 0.305 expected). Together, these results suggest that a juvenile‐heavy composition is not confined to a single sampling method and should be accounted for in any demographic inference drawn from mortality records.

Why might juveniles be over‐represented? Several mechanisms, possibly acting in concert, could explain this pattern. First, juvenile harbour porpoises may experience elevated mortality beyond the baseline MPM parameterisation. Early‐life vulnerability is well documented in marine mammals and other taxa (Cook et al. 1971; Linnell et al. 1995), driven by factors such as immunological immaturity, energetic stress during and shortly after weaning, inexperience in foraging or predator avoidance, and higher susceptibility to disease, contaminants or fisheries interactions. If juvenile mortality is indeed higher than adult mortality, particularly if it is substantially higher, then juveniles will be over‐represented in any mortality sample.

Second, differential detection and recovery probabilities could contribute to juvenile over‐representation. Smaller‐bodied juveniles might drift differently from larger adults, influenced by buoyancy dynamics, wind, and ocean currents, making them more likely to wash ashore in accessible locations. Alternatively, juveniles might be more visible or more readily reported by beachgoers, though we consider this less likely given that body size differences between juveniles and adults are not extreme. A third possibility is that our baseline model, an asymptotic reference derived from assumed vital rates, is misspecified. A higher juvenile share in the living population would need lower juvenile mortality or higher adult mortality than we assumed (or higher fertility), but across the ranges we tested the juvenile share of the living population stayed between 25% and 35%, well below the 58% seen in strandings (Table S13). For the composition of deaths, the effect goes the other way: juveniles would form a larger share of deaths if juvenile mortality were higher, or adult mortality lower, than we assume. Because the composition of deaths is more sensitive to these rates, we compare the data with both the living population and the expected composition of deaths. Our sensitivity analyses with varied vital rates (Tables S11–S14) and with simulated MPMs suggest that the qualitative conclusion that juveniles are over‐represented is robust across a wide range of plausible demographic parameter space, although with the death‐based expectation a minority of parameter sets (3%–8%) can reproduce the observed juvenile share, mainly with low juvenile survival. Matching the strandings would need juvenile survival of about 0.62 (Table S14), well below the value we assumed, so independent estimates of juvenile survival are the key missing information for deciding whether the juvenile excess reflects sampling bias or higher juvenile mortality. If published survival rates are themselves derived from opportunistic samples, they may carry the same bias, and independent estimates of age‐specific mortality are needed.

The magnitude of departure from asymptotic stage structure, as quantified primarily by Keyfitz's Δ (with concordant alternative metrics; Tables S3 and S9), varied considerably across regions and over time. Sustained increases in Regions C and E over the 27‐year study period are consistent with changing fisheries pressure or habitat change altering age‐specific mortality exposure, rather than reflecting static sampling bias alone. This heterogeneity suggests that demographic bias is driven by regional differences in mortality processes, population structure, and environmental conditions rather than a single uniform mechanism. The modest regional synchrony in divergence through time (Figure S4) is also more consistent with region‐specific drivers than with a single basin‐wide forcing acting uniformly across all regions. Regions with particularly high divergence may be experiencing elevated juvenile mortality due to localised stressors (e.g., intensive fisheries activity, habitat degradation, prey depletion), or they may differ in detection and reporting dynamics that selectively filter age classes. Notably, the strongest divergence is concentrated in Regions D–E (English/Belgian/Dutch sectors) rather than Region F, which is nearest the Baltic transition zone, suggesting that spatial variation in mortality composition reflects regional differences in mortality processes, carcass transport, and detection effort rather than a simple Baltic‐proximity gradient.

The bycatch result is notable because it challenges our initial expectation that bycatch would be less demographically biased than strandings. Instead, bycatch was at least as divergent from SSD and, in paired years, often more so. Several non‐exclusive mechanisms could explain this. First, bycatch risk is likely gear‐selective: certain net types, mesh sizes, soak times, and deployment depths may disproportionately intercept younger/smaller porpoises (Kindt‐Larsen et al. 2023). Second, habitat overlap can raise bycatch risk independently of population stage structure: porpoise foraging areas and coastal gillnet effort can co‐occur in prey‐rich shelf habitats, creating local exposure hotspots (Maeda et al. 2021; Brennecke et al. 2021). Third, the bycatch dataset used here pools records across multiple regions, periods and gear contexts, so compositional heterogeneity can accumulate into a strong aggregate juvenile bias. Taken together, these points suggest that the demographic bias documented here is not a property of stranding data specifically, but a feature of opportunistic mortality sampling more broadly.

Strandings remain a valuable source of information on cetacean biology, health and mortality, providing material for necropsy, tissue sampling, contaminant analysis, diet studies, reproductive assessments and genetic work, all of which would be difficult or impossible to obtain from living animals. The present findings concern representativeness, not utility: the issue is not whether stranding data are informative, but whether they can be treated as unbiased demographic samples of living populations. Strandings also offer early warning signals of emerging threats, such as disease outbreaks, harmful algal blooms, fisheries interactions or environmental contaminant spikes. Our results indicate that strandings should not be uncritically treated as unfiltered or demographically representative samples of living populations. When making inferences about population age structure, stage‐specific survival rates, or overall population trends, the systematic biases we have documented must be acknowledged and, where possible, accounted for.

How can this be done? One approach is to use stranding data primarily for surveillance and hypothesis generation rather than for direct demographic parameter estimation. Strandings can alert us to potential problems, such as unusual mortality events, spatial clustering of deaths and temporal pulses in specific age classes, without necessarily providing precise estimates of vital rates or population growth. A second approach is to integrate stranding data with other data sources in formal statistical models that explicitly account for bias. For example, integrated population models (IPMs) can combine stranding records with survey‐based abundance estimates, telemetry data and bycatch records, using hierarchical modelling frameworks to partition observation error, process error and sampling bias. If stranding detection probabilities or stage‐specific vulnerabilities can be estimated or bounded, they can be incorporated as covariates or priors in the modelling framework.

A third and complementary approach, which we have pursued here, is to develop explicit diagnostics that quantify the magnitude and direction of bias. By comparing observed compositions with asymptotic, model‐based expectations, we provide a calibration tool to inform how much weight to place on stranding‐derived demographic inferences. If divergence from asymptotic stage structure (SSD) is modest, strandings may be cautiously used for demographic inference, with appropriate caveats. If divergence is large and persistent, as we found for juveniles, alternative data sources or strong corrective models are needed.

The framework requires only two inputs: age‐composition data (which can be derived from body size if direct age estimates are unavailable) and a demographically informed MPM (which can be parameterised using the literature, data from related species or expert opinion). Both inputs are increasingly available for species of conservation concern.

We emphasise that deviation from SSD should not be interpreted as evidence that strandings are necessarily misrepresenting the true population structure; a living population in a transient demographic state will also depart from SSD independently of any sampling bias. This seems to be the case of the Belt Sea population of porpoises, which has undergone a seemingly drastic decline during the past decade (Owen et al. 2024), although part of this change may also reflect a geographic shift in where porpoises occur. What our framework quantifies is inconsistency between observed mortality composition and asymptotic expectations under the assumed demography, a signal informative about bias, but one that cannot fully separate demographic filtering from genuine transient population dynamics.

This interpretive constraint is one of several limitations worth acknowledging. A potential concern is that the detected deviation from SSD reflects misspecification of the baseline model rather than genuine demographic bias. Our simulation analysis directly addresses this: only 5 of 500 randomly sampled biologically plausible matrices (1.0%) produced SSDs resembling the observed mortality composition under the Keyfitz's Δ ≤ 0.15 criterion. This indicates that the juvenile over‐representation signal cannot readily be attributed to a particular choice of vital rates, and that the conclusion is robust to parametric uncertainty in a way that sensitivity analyses around a single baseline cannot fully establish. Even so, vital rates do vary among populations, fluctuate over time in response to environmental conditions, and are difficult to estimate precisely in long‐lived marine mammals. Future work should ideally integrate direct estimates of vital rates from telemetry studies, mark‐recapture data, or longitudinal monitoring programmes where available. Relatedly, our MPM is parameterised primarily from North Sea‐focused literature, whereas Baltic harbour porpoises are genetically distinct and may have different vital rates; this could influence the absolute magnitude of the inferred mismatch in Region F (the Baltic‐adjacent sector) in particular, although it does not change the broader finding of substantial divergence from the modelled SSD reference.

We also used body length as a proxy for demographic stage. While this is a practical and widely used approach, it is imperfect. Growth rates vary among individuals, and some animals may reach reproductive maturity at sizes outside our defined thresholds. More refined staging, perhaps incorporating additional morphometric or physiological indicators (e.g., tooth wear, reproductive tract development, hormone levels), could improve precision. However, such data are rarely available for large samples of stranded animals, and the body‐length approach we employed is a practical compromise between biological realism and operational feasibility.

We did not explicitly model detection probability, reporting effort or drift dynamics. These factors likely vary across regions and over time, introducing heterogeneity beyond what our framework captures. In addition, the Murphy mixed‐mortality source partially overlaps the IJsseldijk North Sea stranding geography (regions A‐C and part of D), so some cross‐source comparisons are not fully independent. Where available, cause‐of‐death information could also provide important context: strandings due to bycatch might show different demographic patterns from those due to disease or starvation.

In our baseline model the population grows at about 3% a year, below the 5%–10% maximum reported for porpoises, but it is not matched to the local population trend in each region and year, and expected compositions differ between growing and declining populations. We also treat the animals as one randomly mating population, although porpoises show some population structure; if strandings come from groups with different vital rates, the expected composition would differ between areas. Finally, the juvenile period is uncertain: a longer period gives a larger expected juvenile share and a smaller, though still significant, mismatch (Table S17).

## 5 Conclusions

Harbour porpoise mortality records from both strandings and bycatch show substantial and consistent departures from asymptotic demographic expectations, as represented by the stable stage distribution predicted by a stage‐structured matrix population model. Juveniles are significantly over‐represented across all spatial and temporal scales examined relative to asymptotic expectations (the excess is smaller when compared with the expected composition of deaths, and some bycatch, mixed and hunting samples are consistent with it), a pattern that is robust to multiple sensitivity analyses and alternative analytical choices. This departure from the living population has important implications for the interpretation and use of mortality data in demographic inference, risk assessment and conservation planning. Whether the juvenile excess reflects sampling bias or simply higher juvenile mortality cannot be resolved without independent estimates of juvenile survival.

Stranding and bycatch data remain valuable for surveillance, early warning and biological investigation, but they should not be treated as unfiltered or demographically representative samples of living populations. The MPM‐based diagnostic framework we present provides a practical, quantitative tool for assessing representativeness in opportunistic wildlife datasets, including other marine mammal species and other non‐random data streams where demographic bias is suspected but not formally quantified.

Future work should focus on integrating explicit models of detection probability and reporting effort, incorporating covariates such as coastline length, human population density, stranding network coverage and oceanographic conditions (currents and wind), incorporating cause‐of‐death information, and combining stranding data with other demographic data sources in formal integrated population models. In practice, the framework enables more cautious and calibrated use of mortality data in bycatch‐risk assessment and conservation planning.

## Author Contributions

**Michaela Kirstine S. M. H. Søgaard:** conceptualization (equal), data curation (equal), formal analysis (equal), investigation (equal), software (equal), visualization (equal), writing – original draft (equal). **Magnus Wahlberg:** conceptualization (equal), investigation (equal), supervision (equal), writing – review and editing (equal). **Owen R. Jones:** conceptualization (equal), data curation (equal), formal analysis (equal), investigation (equal), methodology (equal), project administration (equal), software (equal), supervision (equal), validation (equal), visualization (equal), writing – original draft (equal), writing – review and editing (equal).

## Funding

The authors have nothing to report.

## Conflicts of Interest

The authors declare no conflicts of interest.

## Supporting information


**Figure S1:** SSD uncertainty distribution from Monte Carlo MPM sampling.
**Figure S2:** Distribution of Keyfitz's Δ across region‐years, showing that SSD divergence is heterogeneous across region‐years.
**Figure S3:** PCA (PC1–PC3) view of life‐history transition‐rate space for unconstrained biologically plausible simulated MPMs, showing the structure of sampled transition combinations in an alternative PCA projection.
**Figure S4:** Pairwise correlation matrix of regional Keyfitz's Δ time series.
**Figure S5:** Strandings population structure through time by region.
**Table S1:** SSD uncertainty summary by stage class, showing that SSD uncertainty intervals are bounded and interpretable across stage classes.
**Table S2:** Summary statistics for Keyfitz's Δ across region‐years, showing that average and upper‐range divergence remain substantial.
**Table S3:** Summary statistics for Hellinger distance across region‐years showing that conclusions are concordant across distance metrics.
**Table S4:** Region‐year descriptive context for stranded harbour porpoises, showing that sampling intensity and temporal coverage vary across regions (A–F).
**Table S5:** Juvenile bias sensitivity across filtering scenarios, showing that the juvenile over‐representation signal is robust to alternative data filters.
**Table S6:** Keyfitz's Δ sensitivity across filtering scenarios, showing that divergence from SSD remains substantial under sensitivity filters.
**Table S7:** Juvenile bias sensitivity to SSD uncertainty bounds, showing that juvenile over‐representation persists under SSD uncertainty.
**Table S8:** Keyfitz's Δ sensitivity to SSD uncertainty bounds, showing that divergence magnitudes remain elevated across plausible SSD variants.
**Table S9:** Chi‐square goodness‐of‐fit results across strandings and bycatch groupings, showing strong and directionally concordant deviation from SSD expectations.
**Table S10:** Observed juvenile percentage and Keyfitz's Δ for each source against the living‐population SSD and the expected composition of deaths.
**Table S11:** Keyfitz's Δ with the model calibrated to a stable population (*λ* = 1).
**Table S12:** Sensitivity of expected stage composition to vital rates (20,000 random parameter sets).
**Table S13:** Effect of changing one vital rate at a time.
**Table S14:** Survival rates that would make the model's death composition match each observed sample.
**Table S15:** Three‐stage model compared with age‐by‐age models with a fixed juvenile period.
**Table S16:** Age‐by‐age share of the living population in the baseline age‐structured model.
**Table S17:** Keyfitz's Δ for each source under the three‐stage and age‐by‐age models.

## Data Availability

Stranding data used in this study are derived from the compiled dataset from IJsseldijk et al. (2020). Bycatch data were sourced from IJsseldijk et al. (2022), Brennecke et al. (2021) and Frie and Lindstrøm (2024); mixed bycatch + stranding mortality data were sourced from Murphy et al. (2020). Historical hunting data were sourced from Lockyer et al. (2001) and Møhl‐Hansen (1954). All analytical code, input data, and derived outputs required to reproduce the analyses are archived on Zenodo: https://doi.org/10.5281/zenodo.18664347.
