# Supplementary material for: Do Harbour Porpoise Mortality Records Reflect Living Population Structure? A Matrix Population Model Diagnostic
Source: Ecol Evol. 2026 Nov 1;16(11):e74607. doi: 10.1002/ece3.74607 (PMC13724302; doi:10.1002/ece3.74607)
Supplement: Supplementary file 1 [file ECE3-16-e74607-s001.docx]

Supplementary Information: Do harbour porpoise mortality records reflect living population structure? A matrix population model diagnostic

2026-10-06

Michaela Kirstine S. M. H. Søgaard^1,2^, Magnus Wahlberg^1,2,3^, and Owen R. Jones^1*^

^1^ Department of Biology, University of Southern Denmark, Odense, Denmark

^2^ Marine Biological Research Centre, University of Southern Denmark, Kerteminde, Denmark

^3^ Fjord & Baelt, Kerteminde, Denmark

^*^ Corresponding author: [jones@biology.sdu.dk](mailto:jones@biology.sdu.dk)

# Supplementary Results

This supplement includes additional material referenced in the main text: SSD uncertainty outputs (Figure S1, Table S1), distribution summaries for divergence metrics (Figure S2, Tables S2-S3), an additional MPM-space PCA view (Figure S3), the regional synchrony correlation matrix (Figure S4), descriptive stranding context (Table S4, Figure S5), sensitivity analyses (Tables S5-S8), and additional goodness-of-fit diagnostics supporting concordance with Keyfitz’s Δ (Table S9).

## Supplementary Methods for secondary diagnostics

In the main manuscript, Keyfitz’s Δ is treated as the primary divergence metric. Here we document the secondary diagnostics used to verify robustness of inference.

Chi-square goodness-of-fit tests were computed as:

$$\chi^{2}=\sum_{i} \frac{(O_{i}-E_{i})^{2}}{E_{i}},$$

where $O_{i}$ and $E_{i}$ are observed and SSD-expected counts for stage $i$.

Hellinger distance was computed as:

$$H=\sqrt{1-\sum_{i} \sqrt{p_{i,obs} p_{i,SSD}}},$$

where $p_{i,obs}$ and $p_{i,SSD}$ are observed and SSD-expected stage proportions. This metric was used as a complementary distance check and interpreted for concordance with Keyfitz’s Δ, not as a replacement for the primary metric.

### Simulation-based plausibility settings

For the simulation-based plausibility analysis, we generated random 3-stage Lefkovitch matrices using mpmsim::rand_lefko_set in code/analysis/porpoise-modeling.R. We used archetype 4 (monotonic forward stage progression without retrogression), output = "Type1", and a fixed random seed (set.seed(43)). We generated 500 matrices (n = 500), constraining only the dominant eigenvalue to a biologically plausible range ($\lambda\in[0.7,1.3]$). Fecundity was given broad variation via the specified fecundity bounds ($F_{A}$ from 0 to 10; other fertility entries fixed to 0). For each simulated matrix, we calculated the SSD and then Keyfitz’s $\Delta$ against the observed overall stranding composition (not the baseline SSD). We report the proportion of simulations meeting the Keyfitz’s Δ resemblance thresholds of ($\Delta\leq0.15$, $\Delta\leq0.10$) and summary statistics of the simulated $\Delta$ distribution.

## SSD uncertainty

Figure S1: SSD uncertainty distribution from Monte Carlo MPM sampling. This shows that plausible SSD stage proportions vary within bounded ranges under vital-rate uncertainty, supporting robustness interpretation in the main manuscript.

Table S1: SSD uncertainty summary by stage class, showing that SSD uncertainty intervals are bounded and interpretable across stage classes.

| **Ageclass** | **n** | **mean** | **median** | **sd** | **CI low** | **CI high** | **min** | **max** |
| --- | --- | --- | --- | --- | --- | --- | --- | --- |
| Adult | 2,000 | 0.495 | 0.493 | 0.036 | 0.425 | 0.568 | 0.384 | 0.683 |
| Juvenile | 2,000 | 0.306 | 0.305 | 0.029 | 0.253 | 0.364 | 0.182 | 0.408 |
| Neonate | 2,000 | 0.200 | 0.200 | 0.017 | 0.163 | 0.233 | 0.136 | 0.259 |

## Distribution of divergence metrics

This section provides the additional metric checks used to verify that conclusions do not depend on Keyfitz’s Δ alone. Hellinger distance follows the same regional and temporal structure as Keyfitz’s Δ, supporting robustness of the main interpretation.

Figure S2: Distribution of Keyfitz’s Δ across region-years, showing that SSD divergence is heterogeneous across region-years.

Table S2: Summary statistics for Keyfitz’s Δ across region-years, showing that average and upper-range divergence remain substantial.

| **n** | **mean** | **median** | **sd** | **min** | **max** |
| --- | --- | --- | --- | --- | --- |
| 167 | 0.268 | 0.257 | 0.122 | 0.02 | 0.695 |

Table S3: Summary statistics for Hellinger distance across region-years showing that conclusions are concordant across distance metrics.

| **n** | **mean** | **median** | **sd** | **min** | **max** |
| --- | --- | --- | --- | --- | --- |
| 167 | 0.228 | 0.224 | 0.107 | 0.017 | 0.669 |

## Additional MPM space visualisation

Figure S3: PCA (PC1–PC3) view of life-history transition-rate space for unconstrained biologically plausible simulated MPMs, showing the structure of sampled transition combinations in an alternative PCA projection. Point colour encodes Keyfitz’s Δ (divergence from observed overall stage composition), and point shape indicates matrix type (simulated vs reference).

## Regional synchrony matrix

Figure S4: Pairwise correlation matrix of regional Keyfitz’s Δ time series. Each cell value is a Pearson correlation coefficient between two regional divergence trajectories over time; higher positive values indicate stronger temporal synchrony in SSD divergence between those regions.

## Descriptive stranding context by region-year

Table S4: Region-year descriptive context for stranded harbour porpoises, showing that sampling intensity and temporal coverage vary across regions (A-F).

| **Region** | **years** | **total strandings** | **median annual n** | **iqr annual n** | **min annual n** | **max annual n** |
| --- | --- | --- | --- | --- | --- | --- |
| A | 1991-2017 | 456 | 16.0 | 7.50 | 2 | 35 |
| B | 1990-2017 | 610 | 20.0 | 15.50 | 4 | 52 |
| C | 1990-2017 | 554 | 17.5 | 8.75 | 7 | 60 |
| D | 1990-2017 | 2,702 | 82.0 | 137.50 | 5 | 300 |
| E | 1990-2017 | 3,846 | 94.5 | 187.75 | 12 | 444 |
| F | 1990-2017 | 2,695 | 85.0 | 69.00 | 36 | 225 |

Figure S5: Strandings population structure through time by region. Each panel represents a region; points are observed annual age-class proportions and coloured lines trace temporal trajectories for neonates, juveniles, and adults, showing how stranding composition changes through time within regions.

## Sensitivity analyses

Table S5: Juvenile bias sensitivity across filtering scenarios, showing that the juvenile over-representation signal is robust to alternative data filters.

| **scenario** | **estimate** | **expected** | **p-value** | **n** |
| --- | --- | --- | --- | --- |
| all region year | 0.581 | 0.305 | <0.001 | 10,863 |
| complete ageclasses | 0.581 | 0.305 | <0.001 | 10,481 |
| min count 10 | 0.581 | 0.305 | <0.001 | 10,778 |
| complete and min 10 | 0.581 | 0.305 | <0.001 | 10,430 |

Table S6: Keyfitz’s Δ sensitivity across filtering scenarios, showing that divergence from SSD remains substantial under sensitivity filters.

| **scenario** | **n** | **mean** | **median** | **sd** | **min** | **max** |
| --- | --- | --- | --- | --- | --- | --- |
| all region year | 167 | 0.268 | 0.257 | 0.122 | 0.02 | 0.695 |
| complete ageclasses | 139 | 0.257 | 0.256 | 0.116 | 0.02 | 0.504 |
| min count 10 | 154 | 0.263 | 0.258 | 0.117 | 0.02 | 0.632 |
| complete and min 10 | 133 | 0.256 | 0.256 | 0.117 | 0.02 | 0.504 |

Table S7: Juvenile bias sensitivity to SSD uncertainty bounds, showing that juvenile over-representation persists under SSD uncertainty.

| **scenario** | **expected** | **p-value** | **n** |
| --- | --- | --- | --- |
| ssd mean | 0.306 | <0.001 | 10,863 |
| ssd ci low | 0.300 | <0.001 | 10,863 |
| ssd ci high | 0.313 | <0.001 | 10,863 |

Table S8: Keyfitz’s Δ sensitivity to SSD uncertainty bounds, showing that divergence magnitudes remain elevated across plausible SSD variants.

| **scenario** | **n** | **mean** | **median** | **sd** | **min** | **max** |
| --- | --- | --- | --- | --- | --- | --- |
| ssd mean | 167 | 0.268 | 0.257 | 0.122 | 0.020 | 0.694 |
| ssd ci low | 167 | 0.272 | 0.262 | 0.123 | 0.020 | 0.700 |
| ssd ci high | 167 | 0.262 | 0.252 | 0.121 | 0.027 | 0.687 |

## Chi-square diagnostics

These diagnostics report formal goodness-of-fit test results for the strandings and bycatch datasets. They are presented here to document statistical agreement with the Keyfitz’s Δ results while keeping the main manuscript focused on the primary distance metric, Keyfitz’s Δ.

Table S9: Chi-square goodness-of-fit results across strandings and bycatch groupings, showing strong and directionally concordant deviation from SSD expectations.

| **dataset** | **group** | **statistic** | **df** | **p-value** | **n** |
| --- | --- | --- | --- | --- | --- |
| Strandings overall | Overall | 3,901.009 | 2 | 0.000 | 10,863 |
| Strandings by region | A | 132.776 | 2 | 0.000 | 456 |
| Strandings by region | B | 257.310 | 2 | 0.000 | 610 |
| Strandings by region | C | 170.561 | 2 | 0.000 | 554 |
| Strandings by region | D | 1,763.331 | 2 | 0.000 | 2,702 |
| Strandings by region | E | 1,957.532 | 2 | 0.000 | 3,846 |
| Strandings by region | F | 293.011 | 2 | 0.000 | 2,695 |
| Strandings by country | Belgium | 810.909 | 2 | 0.000 | 877 |
| Strandings by country | Denmark | 228.721 | 2 | 0.000 | 998 |
| Strandings by country | England | 329.289 | 2 | 0.000 | 1,143 |
| Strandings by country | Germany | 312.937 | 2 | 0.000 | 1,697 |
| Strandings by country | Netherlands | 2,827.688 | 2 | 0.000 | 5,187 |
| Strandings by country | Scotland | 365.316 | 2 | 0.000 | 961 |
| Bycatch overall | Overall | 62.631 | 2 | 0.000 | 270 |
| Bycatch by source | Brennecke 2021 | 118.695 | 2 | 0.000 | 136 |
| Bycatch by source | Frie Lindstrom 2024 | 0.702 | 2 | 0.704 | 134 |

## Death-based expectation, vital-rate sensitivity and age-structured checks

The tables in this section support the Methods and Results in the main text. Table S10 compares the data with both the living-population SSD and the expected composition of deaths. Table S11 repeats this with the model set to a stable population (λ = 1). Tables S12 and S13 show how the expectations change with the vital rates, and Table S14 shows the survival rates that would be needed to match the data. Tables S15–S17 compare the three-stage model with age-by-age models with a fixed juvenile period. All were produced by code/analysis/death-expectation-sensitivity.R, age-structured-check.R and revision-tables.R.

Table S10: Baseline model: observed juvenile percentage and Keyfitz’s Δ for each source against the living-population SSD and against the expected composition of deaths (SSD × stage-specific mortality, rescaled). p-values are from two-sided tests of the juvenile proportion.

| **Source** | **n** | **Observed juvenile %** | **Living SSD: expected juvenile %** | **Living SSD: Keyfitz Δ** | **Living SSD: p (two-sided)** | **Deaths: expected juvenile %** | **Deaths: Keyfitz Δ** | **Deaths: p (two-sided)** |
| --- | --- | --- | --- | --- | --- | --- | --- | --- |
| Strandings: all regions | 10,863 | 58.1 | 30.5 | 0.28 | <0.001 | 33.4 | 0.25 | <0.001 |
| Strandings: region A | 456 | 54.8 | 30.5 | 0.24 | <0.001 | 33.4 | 0.21 | <0.001 |
| Strandings: region B | 610 | 60.0 | 30.5 | 0.29 | <0.001 | 33.4 | 0.27 | <0.001 |
| Strandings: region C | 554 | 55.2 | 30.5 | 0.25 | <0.001 | 33.4 | 0.22 | <0.001 |
| Strandings: region D | 2,702 | 67.7 | 30.5 | 0.37 | <0.001 | 33.4 | 0.34 | <0.001 |
| Strandings: region E | 3,846 | 63.4 | 30.5 | 0.33 | <0.001 | 33.4 | 0.30 | <0.001 |
| Strandings: region F | 2,695 | 41.6 | 30.5 | 0.16 | <0.001 | 33.4 | 0.08 | <0.001 |
| Bycatch: Brennecke 2021 | 136 | 73.5 | 30.5 | 0.43 | <0.001 | 33.4 | 0.40 | <0.001 |
| Bycatch: Frie Lindstrom 2024 | 134 | 31.3 | 30.5 | 0.03 | 0.911 | 33.4 | 0.14 | 0.675 |
| Bycatch: IJsseldijk et al. 2022 | 103 | 72.8 | 30.5 | 0.42 | <0.001 | 33.4 | 0.39 | <0.001 |
| Hunting: Lockyer 2001, Maniitsoq North | 126 | 38.9 | 30.5 | 0.09 | 0.052 | 33.4 | 0.18 | 0.228 |
| Hunting: Lockyer 2001, Nuuk Paamiut | 52 | 38.5 | 30.5 | 0.17 | 0.275 | 33.4 | 0.05 | 0.533 |
| Hunting: Møhl-Hansen 1954 | 389 | 19.3 | 30.5 | 0.31 | <0.001 | 33.4 | 0.43 | <0.001 |
| Mixed: Murphy 2020, Celtic Irish 1990-1999 | 217 | 34.1 | 30.5 | 0.17 | 0.285 | 33.4 | 0.05 | 0.889 |
| Mixed: Murphy 2020, Celtic Irish 2000-2012 | 172 | 50.6 | 30.5 | 0.23 | <0.001 | 33.4 | 0.17 | <0.001 |
| Mixed: Murphy 2020, North Sea 1990-1999 | 155 | 40.0 | 30.5 | 0.17 | 0.013 | 33.4 | 0.07 | 0.099 |
| Mixed: Murphy 2020, North Sea 2000-2012 | 101 | 36.6 | 30.5 | 0.06 | 0.221 | 33.4 | 0.09 | 0.563 |

Table S11: Keyfitz’s Δ with the model calibrated to a stable population (λ = 1), either by lowering fertility or by lowering adult survival (other rates at baseline). The baseline model has λ = 1.03.

| **Source** | **Baseline (λ=1.03): SSD** | **Baseline: deaths** | **λ=1 via fertility: SSD** | **λ=1 via fertility: deaths** | **λ=1 via adult survival: SSD** | **λ=1 via adult survival: deaths** |
| --- | --- | --- | --- | --- | --- | --- |
| Strandings: all regions | 0.28 | 0.25 | 0.31 | 0.27 | 0.27 | 0.30 |
| Strandings: region A | 0.24 | 0.21 | 0.27 | 0.24 | 0.24 | 0.26 |
| Strandings: region B | 0.29 | 0.27 | 0.33 | 0.29 | 0.29 | 0.32 |
| Strandings: region C | 0.25 | 0.22 | 0.28 | 0.24 | 0.25 | 0.27 |
| Strandings: region D | 0.37 | 0.34 | 0.40 | 0.37 | 0.37 | 0.39 |
| Strandings: region E | 0.33 | 0.30 | 0.36 | 0.32 | 0.33 | 0.35 |
| Strandings: region F | 0.16 | 0.08 | 0.23 | 0.11 | 0.16 | 0.13 |
| Bycatch: Brennecke 2021 | 0.43 | 0.40 | 0.46 | 0.42 | 0.43 | 0.45 |
| Bycatch: Frie Lindstrom 2024 | 0.03 | 0.14 | 0.04 | 0.08 | 0.03 | 0.14 |
| Bycatch: IJsseldijk et al. 2022 | 0.42 | 0.39 | 0.45 | 0.42 | 0.42 | 0.44 |
| Hunting: Lockyer 2001, Maniitsoq North | 0.09 | 0.18 | 0.11 | 0.14 | 0.09 | 0.20 |
| Hunting: Lockyer 2001, Nuuk Paamiut | 0.17 | 0.05 | 0.23 | 0.11 | 0.16 | 0.10 |
| Hunting: Møhl-Hansen 1954 | 0.31 | 0.43 | 0.25 | 0.37 | 0.32 | 0.40 |
| Mixed: Murphy 2020, Celtic Irish 1990-1999 | 0.17 | 0.05 | 0.23 | 0.11 | 0.16 | 0.08 |
| Mixed: Murphy 2020, Celtic Irish 2000-2012 | 0.23 | 0.17 | 0.30 | 0.20 | 0.23 | 0.22 |
| Mixed: Murphy 2020, North Sea 1990-1999 | 0.17 | 0.07 | 0.23 | 0.11 | 0.16 | 0.12 |
| Mixed: Murphy 2020, North Sea 2000-2012 | 0.06 | 0.09 | 0.12 | 0.06 | 0.06 | 0.11 |

Table S12: Sensitivity of the expected stage composition to vital rates for strandings (all regions), from 20,000 random parameter sets (Latin hypercube) and subsets with stable-ish population growth. Observed juvenile percentage: 58.1%.

| **Draws kept** | **Number of draws** | **Expected juvenile %, living SSD (range)** | **Expected juvenile %, deaths (range)** | **% of draws reaching observed 58.1% (SSD)** | **% of draws reaching 58.1% (deaths)** | **Smallest Keyfitz Δ (SSD)** | **Smallest Keyfitz Δ (deaths)** |
| --- | --- | --- | --- | --- | --- | --- | --- |
| All draws | 20,000 | 26.3 (14.1–43.5) | 32.6 (1.2–92.0) | 0 | 8.1 | 0.15 | 0 |
| lambda 0.95-1.05 | 10,012 | 26.4 (14.1–41.4) | 33.0 (1.2–79.5) | 0 | 3.9 | 0.17 | 0 |
| lambda 0.98-1.02 | 4,164 | 26.6 (14.6–40.7) | 33.3 (1.2–70.9) | 0 | 3.0 | 0.17 | 0 |

Table S13: Changing one vital rate at a time (all others at baseline): ranges of λ, expected juvenile percentage and Keyfitz’s Δ for strandings (all regions).

| **Parameter** | **Range** | **λ** | **Juvenile % living SSD** | **Juvenile % deaths** | **Keyfitz Δ SSD** | **Keyfitz Δ deaths** |
| --- | --- | --- | --- | --- | --- | --- |
| Pregnancy rate | 0.6–1 | 0.99–1.05 | 26.7–31.4 | 30.4–34.1 | 0.27–0.31 | 0.24–0.28 |
| Juvenile maturation probability | 0.25–0.5 | 1.02–1.05 | 24.8–34.7 | 27.4–37.8 | 0.23–0.33 | 0.20–0.31 |
| Adult survival (neonate survival = adult²) | 0.8–0.98 | 0.97–1.14 | 30.1–30.8 | 25.5–76.4 | 0.27–0.28 | 0.05–0.33 |
| Juvenile survival | 0.6–0.98 | 0.98–1.09 | 28.1–32.6 | 5.5–48.2 | 0.25–0.30 | 0.10–0.53 |
| Neonate survival | 0.6–0.98 | 1.01–1.07 | 27.2–34.5 | 25.2–49.8 | 0.24–0.31 | 0.15–0.33 |

Table S14: Survival rates that would make the model’s death composition match each observed sample (maximum-likelihood fit with λ restricted to 0.97–1.03; fertility and maturation at baseline). Three rates are fitted to two independent proportions, so these values are not unique estimates of mortality.

| **Source** | **n** | **Adult survival** | **Neonate survival** | **Juvenile survival** | **λ** |
| --- | --- | --- | --- | --- | --- |
| Strandings: all regions | 10,863 | 0.89 | 0.87 | 0.62 | 1.01 |
| Strandings: region A | 456 | 0.86 | 0.92 | 0.68 | 1.01 |
| Strandings: region B | 610 | 0.86 | 0.93 | 0.61 | 1.00 |
| Strandings: region C | 554 | 0.84 | 0.92 | 0.67 | 1.00 |
| Strandings: region D | 2,702 | 0.89 | 0.90 | 0.44 | 0.97 |
| Strandings: region E | 3,846 | 0.89 | 0.90 | 0.55 | 1.00 |
| Strandings: region F | 2,695 | 0.87 | 0.76 | 0.72 | 1.01 |
| Bycatch: Brennecke 2021 | 136 | 0.91 | 0.93 | 0.37 | 0.97 |
| Bycatch: Frie Lindstrom 2024 | 134 | 0.78 | 0.83 | 0.83 | 1.00 |
| Bycatch: IJsseldijk et al. 2022 | 103 | 0.90 | 0.94 | 0.39 | 0.97 |
| Hunting: Lockyer 2001, Maniitsoq North | 126 | 0.78 | 0.88 | 0.78 | 0.99 |
| Hunting: Lockyer 2001, Nuuk Paamiut | 52 | 0.86 | 0.71 | 0.71 | 0.99 |
| Hunting: Møhl-Hansen 1954 | 389 | 0.65 | 1.00 | 0.92 | 0.99 |
| Mixed: Murphy 2020, Celtic Irish 1990-1999 | 217 | 0.86 | 0.66 | 0.73 | 0.99 |
| Mixed: Murphy 2020, Celtic Irish 2000-2012 | 172 | 0.90 | 0.78 | 0.64 | 1.02 |
| Mixed: Murphy 2020, North Sea 1990-1999 | 155 | 0.85 | 0.71 | 0.68 | 0.98 |
| Mixed: Murphy 2020, North Sea 2000-2012 | 101 | 0.79 | 0.78 | 0.76 | 0.97 |

Table S15: Three-stage model compared with age-by-age (Lefkovitch) models in which juveniles take a fixed number of years (one-year age classes with an adult plus-group).

| **Model** | **λ** | **Living SSD: neonate / juvenile / adult %** | **Deaths: neonate / juvenile / adult %** |
| --- | --- | --- | --- |
| Three-stage (geometric juvenile stage, mean 3 y) | 1.034 | 20.1 / 30.5 / 49.4 | 28.7 / 33.4 / 37.9 |
| Age-structured, juveniles ages 1-3 (adult from 4) | 1.021 | 19.2 / 34.2 / 46.7 | 27.3 / 37.2 / 35.5 |
| Age-structured, juveniles ages 1-2 (adult from 3) | 1.047 | 21.0 / 26.7 / 52.3 | 30.2 / 29.4 / 40.4 |
| Age-structured, juveniles ages 1-4 (adult from 5) | 1.002 | 17.8 / 39.9 / 42.4 | 25.0 / 43.0 / 32.0 |

Table S16: Age-by-age share of the living population in the baseline age-structured model (juveniles aged 1–3 years, adults from age 4).

| **Age** | **Share of living population %** |
| --- | --- |
| 0 | 19.2 |
| 1 | 14.1 |
| 2 | 11.2 |
| 3 | 8.9 |
| 4+ | 46.7 |

Table S17: Keyfitz’s Δ for each data source under the three-stage and age-by-age models, against the living-population SSD and the expected composition of deaths. 3-stage = baseline; Juv. 1-3 y = juveniles aged 1–3 (adults from age 4); Juv. 1-2 y and Juv. 1-4 y = shorter and longer juvenile periods.

| **Source** | **3-stage SSD** | **3-stage deaths** | **Juv. 1-3 y SSD** | **Juv. 1-3 y deaths** | **Juv. 1-2 y SSD** | **Juv. 1-2 y deaths** | **Juv. 1-4 y SSD** | **Juv. 1-4 y deaths** |
| --- | --- | --- | --- | --- | --- | --- | --- | --- |
| Strandings: all regions | 0.28 | 0.25 | 0.24 | 0.21 | 0.31 | 0.29 | 0.18 | 0.15 |
| Strandings: region A | 0.24 | 0.21 | 0.21 | 0.18 | 0.28 | 0.25 | 0.15 | 0.16 |
| Strandings: region B | 0.29 | 0.27 | 0.26 | 0.23 | 0.33 | 0.31 | 0.20 | 0.18 |
| Strandings: region C | 0.25 | 0.22 | 0.21 | 0.20 | 0.29 | 0.26 | 0.15 | 0.17 |
| Strandings: region D | 0.37 | 0.34 | 0.34 | 0.31 | 0.41 | 0.38 | 0.28 | 0.25 |
| Strandings: region E | 0.33 | 0.30 | 0.29 | 0.26 | 0.37 | 0.34 | 0.24 | 0.20 |
| Strandings: region F | 0.16 | 0.08 | 0.14 | 0.04 | 0.19 | 0.12 | 0.09 | 0.01 |
| Bycatch: Brennecke 2021 | 0.43 | 0.40 | 0.39 | 0.36 | 0.47 | 0.44 | 0.34 | 0.31 |
| Bycatch: Frie Lindstrom 2024 | 0.03 | 0.14 | 0.05 | 0.16 | 0.05 | 0.13 | 0.09 | 0.19 |
| Bycatch: IJsseldijk et al. 2022 | 0.42 | 0.39 | 0.39 | 0.36 | 0.46 | 0.43 | 0.33 | 0.30 |
| Hunting: Lockyer 2001, Maniitsoq North | 0.09 | 0.18 | 0.08 | 0.16 | 0.12 | 0.19 | 0.08 | 0.18 |
| Hunting: Lockyer 2001, Nuuk Paamiut | 0.17 | 0.05 | 0.14 | 0.03 | 0.20 | 0.09 | 0.11 | 0.05 |
| Hunting: Møhl-Hansen 1954 | 0.31 | 0.43 | 0.34 | 0.45 | 0.28 | 0.40 | 0.38 | 0.49 |
| Mixed: Murphy 2020, Celtic Irish 1990-1999 | 0.17 | 0.05 | 0.14 | 0.06 | 0.20 | 0.08 | 0.15 | 0.09 |
| Mixed: Murphy 2020, Celtic Irish 2000-2012 | 0.23 | 0.17 | 0.20 | 0.13 | 0.26 | 0.21 | 0.16 | 0.08 |
| Mixed: Murphy 2020, North Sea 1990-1999 | 0.17 | 0.07 | 0.14 | 0.03 | 0.19 | 0.11 | 0.09 | 0.03 |
| Mixed: Murphy 2020, North Sea 2000-2012 | 0.06 | 0.09 | 0.03 | 0.08 | 0.10 | 0.10 | 0.03 | 0.12 |
